# Proposed simplified methodological approach for designing geopolymer concrete mixtures

**DOI:** 10.1038/s41598-024-66093-y

**Published:** 2024-07-02

**Authors:** George Uwadiegwu Alaneme, Kolawole Adisa Olonade, Ebenezer Esenogho, Mustapha Muhammad Lawan

**Affiliations:** 1 Civil Engineering Department, Kampala International University, Kampala, Uganda; 2 Department of Electrical, Telecommunication and Computer Engineering, Kampala International University, Kampala, Uganda; 3 Department of Electrical Engineering Science (Centre for Telecommunication), University of Johannesburg, Auckland Park, South Africa; 4 Department of Electrical Engineering, University of Botswana, Gaborone, Botswana

**Keywords:** Geopolymer concrete, Mix design methodology, Agro-industrial waste, Controlling factors, Engineering, Materials science

## Abstract

The development of geopolymer concrete offers promising prospects for sustainable construction practices due to its reduced environmental impact compared to conventional Portland cement concrete. However, the complexity involved in geopolymer concrete mix design often poses challenges for engineers and practitioners. In response, this study proposes a simplified approach for designing geopolymer concrete mixtures, drawing upon principles from Portland cement concrete mix design standards and recommended molar ratios of oxides involved in geopolymer synthesis. The proposed methodology aims to streamline the mix design process while optimizing key factors such as chemical composition, alkali activation solution, water content, and curing conditions to achieve desired compressive strength and workability. By leveraging commonalities between Portland cement concrete and geopolymer concrete, this approach seeks to facilitate the adoption of geopolymer concrete in practical construction applications. The proposed mix design guidelines have been validated through examples for concrete cured under different conditions, including outdoor and oven curing. Future research should focus on validating the proposed methodology through experimental studies and exploring cost-effective alternatives for alkali activation solutions to enhance the feasibility and scalability of geopolymer concrete production. Overall, the proposed simplified approach holds promise for advancing the utilization of geopolymer concrete as a sustainable alternative in the construction industry.

## Introduction

Geopolymer concrete, known for its sustainable and durable properties, offers an environmentally friendly alternative to traditional Portland cement-based concrete. Geopolymer concrete has emerged as a promising alternative to conventional Portland cement-based concrete due to its enhanced sustainability and durability^1^. However, the design process for geopolymer concrete mixes can be intricate due to the need to balance various raw materials and chemical reactions involved in the geopolymerization process, involving complex chemical reactions and material considerations. In response to this complexity, a proposed simplified methodology is introduced to streamline the process of geopolymer concrete mix design^2,3^.

From an environmental perspective, there has been a significant increase in carbon dioxide (CO_2_) emissions due to factors such as energy consumption, transportation, and industrial activities. Cement, while crucial for infrastructure construction, contributes substantially to CO_2_ emissions, with statistics indicating that the production of one ton of cement results in the release of approximately one ton of CO_2_. Consequently, there has been a growing interest in geopolymers as an alternative approach to mitigate CO_2_ emissions associated with cement processing^4,5^.

The concept of geopolymer chemistry was patented by the Geopolymer Institute in 1979, laying the foundation for the development of novel binder materials. Subsequently, in 1983, Joseph Davidovits and James Sawyer introduced high strength geopolymer cement, which marked a significant advancement in the field^6^. Geopolymer binders can be sourced from natural or synthetic aluminosilicates, and the process of geopolymerization involves a chemical reaction between aluminosilicate oxides (known as precursors) and alkali polysilicates, resulting in the formation of polymeric (Si–O–Al) bonds and the creation of amorphous to semi-crystalline three-dimensional silicoaluminate structures^7,8^.

**Figure 1:**
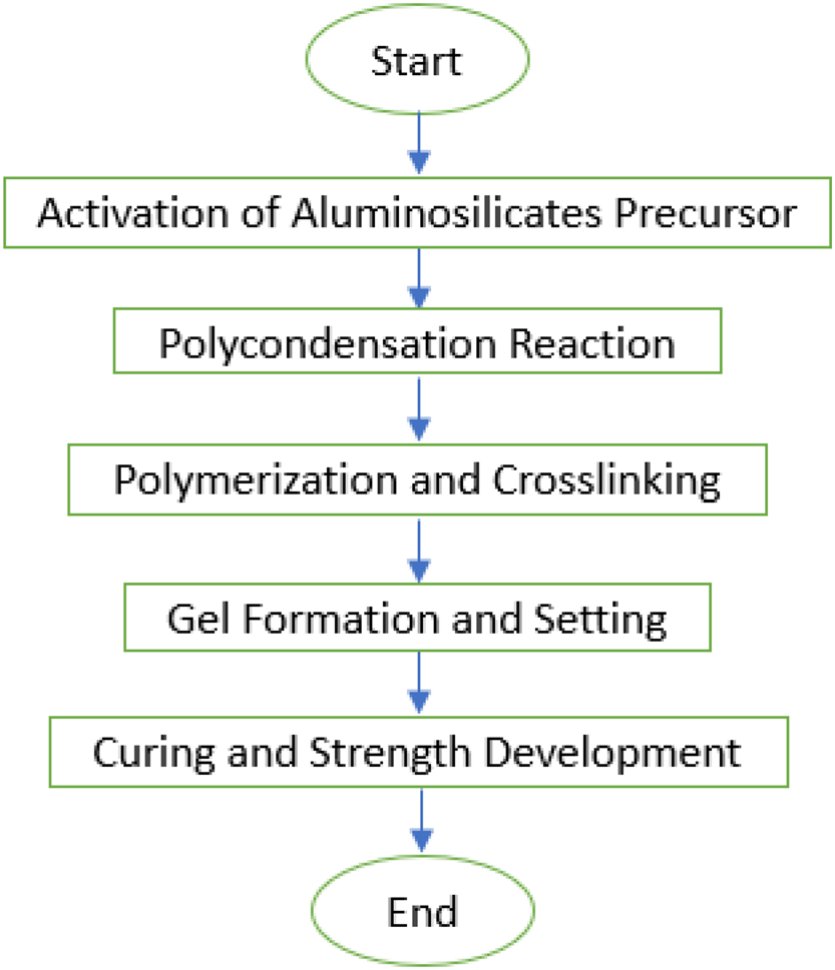
Reaction mechanism of geopolymer^10^.

One notable aspect is that many waste materials contain silica and alumina, making them suitable candidates for use in geopolymerization reactions and as binder materials. the incorporation waste materials into the construction industry, there is potential to enhance both the sustainability and economic viability of infrastructure systems^9,10^. The reaction mechanism of geopolymer can be shown in Fig. 1. The geopolymerization process involves the activation of aluminosilicate precursors through an alkaline activator solution, leading to polycondensation reactions between dissolved silica and alumina species^11^. This results in the formation of polymeric chains consisting of Si–O–Al linkages, which further cross-link to create a three-dimensional network structure. Gel formation occurs, followed by curing, leading to the development of mechanical properties such as compressive strength and durability. Overall, geopolymerization offers a sustainable alternative to traditional cement-based materials, utilizing waste materials and reducing carbon dioxide emissions^12^.

This methodology aims to provide a structured and accessible framework for engineers and practitioners involved in concrete production. By emphasizing key principles and optimizing material selection, mixture proportions, and testing protocols, this approach seeks to simplify the design process while maintaining the performance and sustainability benefits of geopolymer concrete^13,14^. Additionally, we present a comprehensive outline of the proposed methodology, highlighting its goals, essential elements, and possible applications within the construction sector. Through the provision of a simplified method for geopolymer concrete mix design, this approach intends to promote the uptake of sustainable construction methods and support the development of eco-friendly building materials^15^.

This research integrates ACI 211 standards with specific oxide molar ratios to create a simplified mix design methodology for geopolymer concrete. It combines conventional concrete mix design principles with the chemical requirements for geopolymer synthesis, optimizing both mechanical properties and chemical characteristics. The methodology offers a clear, standardized framework, making geopolymer concrete design more accessible. It focuses on optimizing key factors like alkali activation solutions and curing conditions, ensuring practical applicability and cost-effectiveness. This approach facilitates the adoption of geopolymer concrete in the construction industry by aligning with familiar standards and emphasizing experimental validation^16,17^. The aim of this research study is to streamline the design process, emphasize key principles, optimize material selection and mixture proportions, standardize testing protocols, promote sustainable practices, and facilitate industry applications. This approach aims to make geopolymer concrete mix design simplified, more practical, and environmentally friendly for engineers and practitioners in the construction sector.

## Chemical composition and synthesis

Geopolymer concrete is a novel construction material synthesized through a detailed process involving specific raw materials and chemical reactions. Geopolymer concrete is synthesized using aluminosilicate-rich materials, such as agro-industrial wastes, mixed with an alkaline activator solution containing sodium hydroxide and sodium silicate. This mixture undergoes polymerization and cross-linking reactions, forming a three-dimensional network structure of silicoaluminate bonds. As the material sets, it solidifies into a rigid matrix. Curing further enhances its mechanical properties^18^. Overall, geopolymer concrete offers a sustainable alternative to traditional cement-based concrete, with comparable or superior performance characteristics. The following general formula describes the chemical composition as shown in Eq. (1)^19^.

1 $$ {\text{M}}_{{\text{n}}} \left[ {{-}\left( {{\text{SiO}}_{{2}} } \right)_{{\text{Z}}} {-}{\text{AlO}}_{{2}} } \right]_{{\text{n}}} \cdot {\text{wH}}_{{2}} {\text{O}} $$

where M is an alkali cation; z is an integer; n is the degree of polymerization and w is the molar amount of water^7^. The chemistry matrix is a function of four variables, namely: Si/Al ratio, alkali activator type and concentration, curing temperature, and water content.

### Influence of Si/Al ratio

Geopolymers possess a fundamental structure comprised of (SiO_4_) and (AlO_4_) tetrahedrons interconnected by shared oxygen atoms. The Si/Al ratio in geopolymer concrete is a crucial factor that influences various properties and performance aspects of the material. The Si/Al ratio, reflecting this arrangement, significantly influences the behavior of geopolymers. This ratio is inherent to the base material used in geopolymer production^20^. While Si–O–Si bonds are stronger than Al–O–Si bonds, optimal geopolymer performance is achieved with an intermediate Si/Al ratio within a specific alkalinity range. The ideal Si/Al ratio varies depending on the base material and processing conditions. Certain silicates, like those in quartz, may not actively participate in reactivity, emphasizing the importance of the amorphous component as the reactive compound^21^. A higher Si/Al ratio typically leads to faster geopolymerization kinetics and the formation of a denser, more polymerized network structure. This results in improved mechanical properties such as compressive strength and stiffness, enhanced chemical resistance, lower thermal conductivity, and reduced shrinkage and creep^22^. However, excessively high Si/Al ratios may lead to brittleness. Therefore, optimizing the Si/Al ratio is essential to achieve the desired balance of properties for specific application requirements in geopolymer concrete. An increase in the concentration of alumina and silica accelerates the geopolymerization process within the range of 3.20–3.70^23^. However, as the alumina concentration in the mixture increases, neither the formation of the zeolitic phase nor the strength of the samples improves, as noted by Brew et al.^24^. The setting time of the mixture is significantly influenced by the amount of alumina present, with higher ratios of Si to Al resulting in longer setting times. Moreover, an increase in the concentration of alumina leads to a reduction in the strength of the concrete^25^.

### Influence of alkali solution on geopolymer concrete

In general, hydroxide and silicate-based solutions are commonly employed either individually or in various proportions for the synthesis of geopolymers. The composition and concentration of alkali solutions, including hydroxide, silicate-based, and water, significantly influence the performance of geopolymers^26^. Typically, sodium silicate (Na_2_SiO_3_), comprising sodium oxide (Na_2_O), silica dioxide (SiO_2_), and water (H_2_O), is utilized as a silicate-based solution, and can be mixed proportionally with sodium hydroxide (NaOH), potassium hydroxide (KOH), or a combination of both^27^. The activator parameters for sodium silicate are determined by the silica modulus (Ms) or the Na_2_O content, with the silica modulus representing the molar ratio of SiO_2_ to Na_2_O, and the Na_2_O content expressed as a percentage of the weight of the raw material in its dry state. Increasing these parameters decreases the porosity of the mixtures, thereby improving density and maximizing compressive strength values^28^. The concentration of hydroxyl ions can be measured in terms of molarity, with the optimal concentration of NaOH dependent on the curing temperature. For geopolymers containing agro-industrial waste, the NaOH concentration significantly influences the geopolymerization process and impacts the mechanical and physical properties. A low concentration of NaOH in the alkali solution leads to the dissolution of calcium, facilitating the formation of CSH (calcium silicate hydrate) gel, resulting in homogeneous and dense products^27,29^. Conversely, a high NaOH dosage promotes the formation of calcium hydroxide, inhibiting the formation of CSH gel. In this scenario, variable parameters include the weight ratio of low-calcium to high-calcium raw materials and the molar ratio of Na_2_O to SiO_2_. Additionally, unburnt carbon acts as an inert particulate, increasing the demand for activation solution due to absorption. Mechanical activation has recently been investigated as a partial or full replacement for chemical activation in certain geopolymers, yielding promising results and achieving high compressive strength values when used in conjunction with activators^30^.

### Influence of curing mode on geopolymer concrete

The optimization of geopolymer properties is significantly influenced by the curing temperature due to water evaporation. However, excessively high curing temperatures can be detrimental and destabilize the geopolymerization process^31^. Typically, a heat-curing regime is predominantly utilized in geopolymer applications, comprising two main components. Firstly, the curing time ranges from 4 to 96 h, with an optimal practical duration of 24 h. Secondly, the temperature ranges from a minimum of 30 °C to a maximum of 90 °C. Curing methods include steam-curing, curing in covered molds, or dry-curing, each of which affects total porosity, average pore diameter, and microstructural characteristics^32^. Interestingly, Ground Granulated Blast Furnace Slag geopolymers can be optimized at lower curing temperatures compared to low calcium geopolymers^33^. It's worth noting that there is flexibility within the heat-curing regime. For instance, the curing process can be postponed for up to five days without degradation. In precast concrete, there may be instances where molds need to be removed before the completion of the curing time for reuse in another casting, leading to a two-stage curing process. While this flexibility is beneficial for practical purposes, full curing outside the molds remains a subject of debate^34^.

### Influence of water content

The impact of water content is quantified by a single parameter known as the water-to-geopolymer solids ratio by mass. This parameter significantly influences both the compressive strength and workability of geopolymer concrete. The total water mass comprises the combined mass of water in the sodium silicate solution, the water used to produce the sodium hydroxide solution, and any additional water required, if applicable^35^. Conversely, the geopolymer solids mass encompasses the dry raw materials and the solids present in the activator solution, such as those in the sodium hydroxide and sodium silicate solutions (Na_2_O and SiO_2_). Increasing the water-to-geopolymer solids ratio enhances the workability of the concrete^36^. However, there exists an optimal value for this ratio to achieve maximum compressive strength while maintaining acceptable workability. This optimal value is influenced by the type of raw materials and the activator used^37^.

## Geopolymer concrete (GPC)

**Figure 2:**
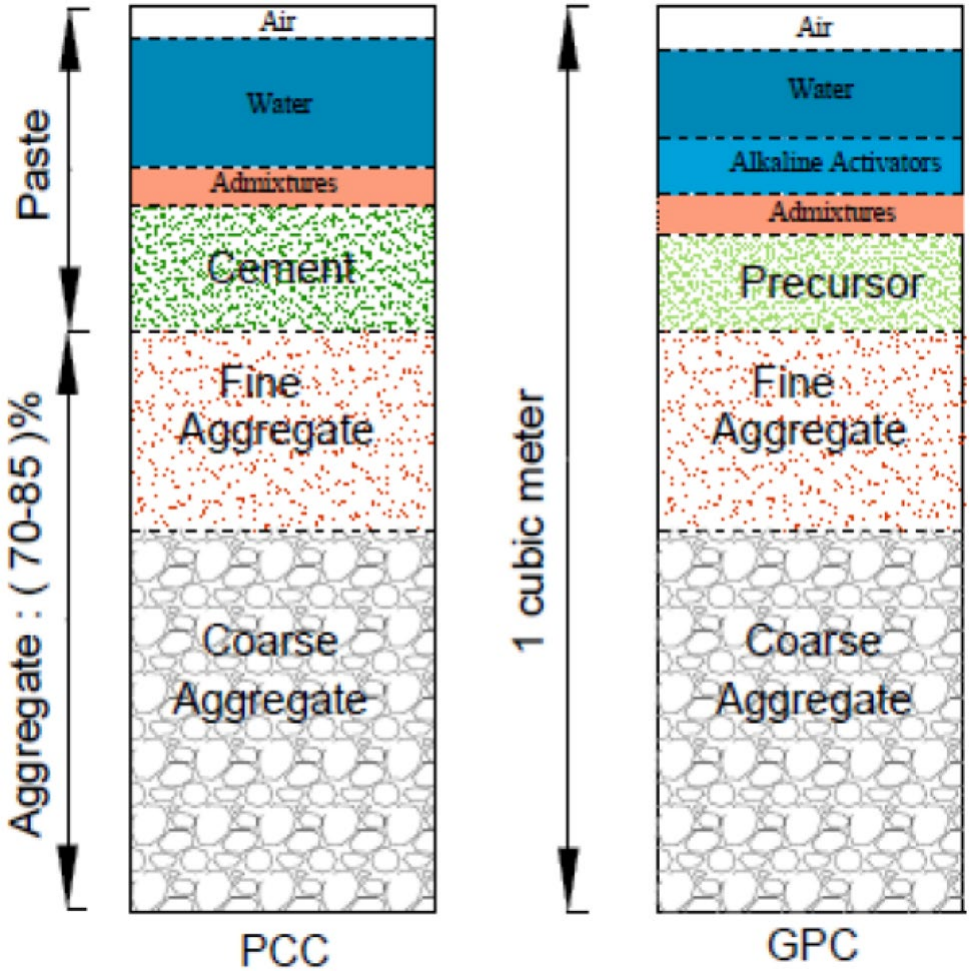
Assessing the Properties of 1m^3^ of PCC and GPC^37^.

The main difference between geopolymer concrete (GPC) and conventional Portland cement-based concrete lies in their binder materials and chemical processes. In conventional Portland cement concrete (PCC), Portland cement acts as the binder, forming calcium silicate hydrate (C–S–H) gel through hydration reactions with water^38^. On the other hand, geopolymer concrete (GPC) uses aluminosilicate-rich materials activated with alkaline solutions to form a geopolymer binder through polymerization reactions. This geopolymer binder results in denser microstructures and reduced porosity, leading to enhanced mechanical properties and durability compared to PCC^39^. This distinction leads to variations in properties such as strength, durability, and environmental impact. However, the conventional methods that are used in the production of Portland cement concrete (PCC) can be utilized to produce geopolymer concrete. Figure 2 shows a typical description of one cubic meter of the volume of Portland cement concrete and geopolymer concrete^40^. GPC also offers environmental benefits by utilizing industrial by-products, reducing carbon dioxide emissions and improved resistance to chemical attack compared to conventional concrete. Overall, while both materials are essential in construction, GPC presents a promising alternative with improved performance and sustainability characteristics^41,42^.

## Proposed simplified method of geopolymer concrete mix design

A simplified mix design methodology is proposed, integrating principles from the ACI 211^43^ standard with recommended molar ratios of oxides crucial for geopolymer synthesis. This approach aims to achieve a desired compressive strength while ensuring workability falls within an acceptable range, as per the standards outlined in ACI 211^43^. The mix design draws parallels between Portland cement concrete and geopolymer concrete mixtures, considering the unique properties of geopolymer concrete. By harmonizing these methodologies, an efficient and effective mix design process can be established for geopolymer concrete applications^44,45^.

### Water content

The water content for geopolymer concrete is a critical factor that influences the workability, strength, and durability of the final product. The amount of water required in geopolymer concrete mixtures depends on several factors, including the characteristics of the raw materials, the desired properties of the concrete, and the specific mix design^46,47^. Generally, the water-to-geopolymer solids ratio is used to determine the water content in geopolymer concrete mixtures. This ratio represents the mass of water divided by the mass of geopolymer solids (including both dry raw materials and solids in the activator solution). The water content in geopolymer concrete should be optimized to achieve a balance between workability and strength. Insufficient water can result in a stiff and difficult-to-handle mixture, leading to poor compaction and decreased strength^48^. On the other hand, excess water can cause segregation, bleeding, and reduced strength due to increased porosity. To determine the appropriate water content for geopolymer concrete, it is essential to conduct mix design trials, considering factors such as the type and proportion of raw materials, the activator solution concentration, and the desired properties of the hardened concrete^49^. Trial mixes should be evaluated for workability, consistency, and strength to identify the optimal water-to-geopolymer solids ratio for the specific application. Additionally, adjustments to the water content may be necessary based on environmental conditions, such as temperature and humidity, during mixing and curing^50^. According to ACI 211^43^ standard, the maximum water content can be determined from the maximum size of aggregate, as is shown in Table 1.

**Table 1:** Estimated water and air content Specifications for various slumps and maximum aggregate sizes in non-air-entrained PCC^43^.

Slump	**Water quality in kg/m**^**3**^** for the nominal maximum aggregate size (mm)**							
	9.5	12.5	19	25	37.5	50	75	100
25–50	207	199	190	179	166	154	130	113
75–100	228	216	205	193	181	169	145	124
150–175	243	228	216	202	190	178	160	–
Entrapped air (%)	3	2.5	2	1.5	1	0.5	0.3	0.2

### Alkaline activator solution content

The alkaline activator solution content is a critical parameter in geopolymer concrete mix design, composed primarily of alkalis like sodium hydroxide and sodium silicate. It initiates the geopolymerization reaction, influencing factors such as reaction kinetics and bond strength. The content varies based on factors like alkali type/concentration, ratio to geopolymer precursors, water content, and project requirements^51^. Proper determination involves mix design trials to achieve desired properties like workability and strength. If no additional water is required for the mixture, the water content is solely derived from the alkaline activator solution^52^. According to Heath et al.^53^, mix oxide molar ratios can be utilized for geopolymer production when employing sodium or potassium hydroxide and silicate (Na_2_O.nSiO_2_ or K_2_O.nSiO_2_) activators, as outlined in Table 2, where M represents Na or K. The choice of alkaline solution will be based on molarity and concentration, adjusted according to the desired water content. Should the selected alkaline solution necessitate less water, any remaining amount required will be added to the mixture as extra water^54^.

**Table 2:** Alkali activator molar ratios for mixing oxides^43^.

Oxide ratio	SiO_2_:Al_2_O_3_	M_2_O:SiO_2_	H_2_O:M_2_O	M_2_O:Al_2_O_3_
Molar ratio range	3.5–4.5	0.2–0.28	15–17.5	0.8–1.2

M represents either sodium (Na) or potassium (K).

### Water-to-geopolymer solids ratio

The water-to-geopolymer solids ratio is a crucial parameter in geopolymer concrete mix design, representing the ratio of water mass to the mass of geopolymer solids. This ratio is essential for determining the proper amount of water needed to achieve the desired workability and strength in the concrete mixture^44^. The water-to-geopolymer solids ratio is typically expressed as a numerical value or percentage, indicating the amount of water relative to the mass of geopolymer precursors (such agro-industrial waste) and the solids present in the activator solution^55^. Achieving the optimal ratio involves balancing workability and mechanical properties through careful consideration and mix design trials. Higher ratios enhance workability but may compromise strength and durability, necessitating a balance tailored to project requirements and environmental conditions. Optimization of this ratio is essential for producing high-quality geopolymer concrete with desired performance characteristics^56^.

**Figure 3:**
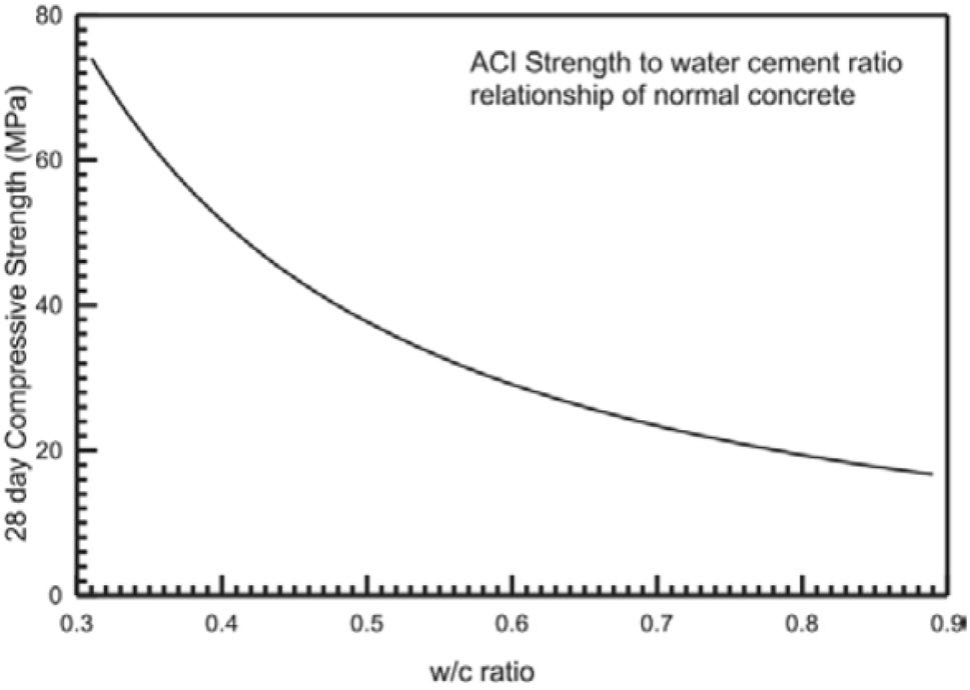
Curve depicting the relationship between strength and water-to-cement ratio^43^.

In PCC, the water-to-cement ratio is determined based on the compressive strength at 28 days, as specified by the ACI 211^43^ standard. Likewise, the water-to-geopolymer solids ratio can be chosen using the standard water-to-cement ratio curve presented in Fig. 3 and Table 3.

**Table 3:** Relationship between water-cement ratio and compressive strength of Portland cement concrete, according to ACI 211^43^ standard.

28 days Compressive strength (MPa)	41	35	28	21	14
Water-cement ratio	0.41	0.48	0.57	0.68	0.82

### Aluminosilicate precursor content

Geopolymer precursor content denotes the quantity of raw materials employed in creating geopolymer concrete. These materials, often agro-industrial waste with aluminosilicate content, are key constituents that undergo geopolymerization to create the concrete's binding matrix. Precursor content significantly impacts concrete properties and performance, influencing factors like strength, workability, and durability^57,58^. Higher precursor content can enhance strength but may reduce workability and increase shrinkage. Adjustments to precursor content are made to achieve desired characteristics, ensuring optimal performance in specific applications and are crucial for producing high-quality geopolymer^59^.

Once the water content and water-to-geopolymer solids ratio (W/GS) have been established, the geopolymer solids content (GS) can be computed using the method outlined in Eqs. (2–5).

2 $$GS=\frac{{W}_{content}}{W/GS}$$

3 $${GS}_{SS}={m}_{ss}\times \%{GS}_{SS}$$

4 $${GS}_{SH}={m}_{sH}\times \%{GS}_{SH}$$

5 $$GS={GS}_{B}+{GS}_{SS}+{GS}_{SH}$$

where GS is geopolymer solid content; GS_SS_ is solid content of Na_2_SiO_3_; GS_SH_ solid content of NaOH; m_SS_ is the content of Na_2_SiO_3_ solution; m_SH_ is content of NaOH solution; GS_B_ is raw material content.

### Entrapped air content volume

The entrapped air content volume in geopolymer concrete refers to the volume of air pockets unintentionally trapped within the mixture during production. Similar to traditional concrete, excessive entrapped air can weaken the material and cause defects. Measuring and controlling this volume is essential for ensuring desired strength, durability, and workability^60^. Techniques such as proper mixing, equipment calibration, and optimizing raw materials are vital for minimizing entrapped air content volume and producing high-quality geopolymer concrete structures. Table 1 displays the percentage of entrapped air in PCC, varying with the maximum aggregate size. However, trials with agro-industrial-based geopolymer have shown higher air content compared to conventional concrete with the same coarse aggregate size^61^. According to ACI 211^43^, a maximum coarse aggregate size of 19 mm corresponds to a 2% air content volume percent. Conversely, for agro-industrial waste-based geopolymer with a maximum coarse aggregate size of 20 mm, the air content volume percent was found to be 3.29^62^. This discrepancy suggests that geopolymer concrete typically has a higher entrapped air percentage than conventional concrete. In this proposed method, the entrapped air content in geopolymer concrete is assumed to be 3.29 V% based on the findings of Ferdous et al.^63^.

### Superplasticizer content in geopolymer

The superplasticizer content in geopolymer concrete refers to the amount of superplasticizer additive added to enhance the workability and flowability of the mixture. It helps overcome the low workability inherent in geopolymer binders by reducing water content and improving particle dispersion. The optimal superplasticizer content depends on factors like the type of superplasticizer, binder characteristics, desired workability, and ambient conditions^64^. Careful selection and dosing are crucial to avoid issues and ensure optimal performance of the concrete. Geopolymer concrete inherently possesses greater stiffness and stickiness compared to conventional concrete. Consequently, using the same water content in geopolymer concrete would result in reduced workability. To enhance workability, options include increasing water content or incorporating superplasticizers like carboxylic ether polymer-based or naphthalene-based types^65^. However, augmenting water content has a more detrimental impact on geopolymer concrete strength compared to incorporating superplasticizers. Hence, adding superplasticizers is a preferable approach to improving geopolymer concrete workability. The recommended dosage of superplasticizer typically falls within the range of 0.8 to 1.5% of the binder content^66^.

### Geopolymer coarse aggregate volume

This refers to the quantity of coarse aggregates incorporated into geopolymer concrete mixtures. These aggregates, typically gravel or crushed stone, contribute to the concrete's strength, durability, and workability. They also help reduce costs and improve dimensional stability. The volume of coarse aggregates is carefully determined based on desired concrete properties and specific project requirements^67^. Overall, their inclusion is essential for optimizing geopolymer concrete performance in various construction applications. As per the ACI 211^43^ standard, the selection of coarse aggregate volume is based on two factors: the nominal maximum size of coarse aggregate and the fineness modulus of fine aggregate, as detailed in Table 4. It's important to highlight that the determination of coarse aggregate volumes follows the method of oven-dry-rodded weights outlined in ASTM C29^43,68^.

**Table 4:** Coarse aggregate volume in 1 cubic meter of PCC^43^.

Nominal maximum aggregate size (mm)	Fineness modulus of fine aggregate			
	2.4	2.6	2.8	3
9.5	0.5	0.48	0.46	0.44
12.5	0.59	0.57	0.55	0.53
19	0.66	0.64	0.62	0.6
25	0.71	0.69	0.67	0.65
37.5	0.75	0.73	0.71	0.69
50	0.78	0.76	0.74	0.72

### Geopolymer fine aggregate content

Geopolymer fine aggregate content refers to the proportion of fine aggregates, such as sand or crushed stone dust, used in geopolymer concrete mixtures. Fine aggregates fill voids, enhance compactness, improve workability, and contribute to strength and dimensional stability. Mix designs carefully balance fine aggregate content to achieve desired performance characteristics^69,70^. Overall, the inclusion of fine aggregates is crucial for optimizing geopolymer concrete properties for various construction applications. Once the volumes of all other ingredients are established, the remaining percentage represents the volume percentage of fine aggregate^43^.

### The aggregates’ moisture content

The moisture content of aggregates is a key factor in concrete production, influencing workability, strength, and durability. Excess moisture can lead to reduced workability and weakened concrete, while insufficient moisture hinders hydration and strength development. Moisture content is measured through various methods and affected by factors such as weather conditions, storage, and aggregate type^71^. Proper control and management, including storage practices and quality control measures, are essential to ensure consistent and high-quality concrete mixes. The moisture content of aggregates impacts two factors: the weight of the aggregates and the amount of mixing water required. Adjustments to the aggregate weight and mixing water content are determined by the saturation level of the batched aggregates^43^.

## Mixing, casting and compacting of geopolymer concrete

The process of mixing, casting, and compacting geopolymer concrete involves thorough mixing of dry ingredients followed by the preparation of alkaline activator solutions. Wet mixing combines the activator solution with the dry mixture, ensuring proper blending and activation of the geopolymer reaction^72^. During casting, formwork is prepared, and the concrete is poured into place, with consolidation techniques used to eliminate voids. Compaction, either mechanical or manual, further enhances density and strength. Proper attention to each step is crucial for ensuring the quality and durability of geopolymer concrete structures^73,74^. One of the defining features of geopolymer concrete lies in its alkaline activator solution, commonly comprised of sodium hydroxide and sodium silicate. Sodium hydroxide solution is prepared by dissolving sodium hydroxide pellets in distilled water and should be shielded from atmospheric exposure for at least 24 h to prevent potential reactions with atmospheric carbonate^75^. Sodium silicate solution, often used in conjunction with sodium hydroxide, can be obtained from manufacturers in specific concentrations. This solution is prepared by dissolving sodium silicate in sodium hydroxide to achieve the desired concentration, with a minimum 24-h preparation period to ensure equilibrium^76,77^.

Alternatively, the addition of amorphous silica with sodium hydroxide can substitute for sodium silicate, as the alkali activator is the costliest component in geopolymer concrete. Once the activator solution is prepared, dry materials and aggregates are mixed for at least three minutes before adding the alkaline liquid, which has been pre-mixed with superplasticizer and any required additional water^78,79^. Wet mixing should continue for a minimum of four minutes. Fresh concrete remains workable for up to 120 min after mixing. Unlike Portland cement concrete (PCC), where dry materials are initially mixed followed by the addition of activator solution, in geopolymer mortar or concrete, the liquid gel (alkali activator solution + precursor + superplasticizer) is formed first before adding and mixing the aggregates. Conducting trial mixes before main experiments is crucial. Compaction procedures for geopolymer concrete mirror those used for conventional concrete^80^.

### Steps in the preparation of agro waste-based geopolymer concrete

Agro waste-based geopolymer concrete refers to a type of concrete that utilizes agricultural waste materials, such as rice husk ash, sugarcane bagasse ash, wheat straw ash, banana peel, and others, as a partial or complete replacement for traditional cementitious materials like Portland cement. The process of producing agro waste-based geopolymer concrete involves converting these agricultural by-products into reactive materials through pre-processing techniques like drying and grinding^81,82^. The required preparation steps are:

- Agro Waste Selection: Choose suitable agro waste materials such as rice husk ash, sugarcane bagasse ash, wheat straw ash, banana peel or any other appropriate waste material.
- Pre-processing: Process the agro waste by drying it to remove moisture and then grinding it into a fine powder. This step ensures uniform particle size and improves reactivity.
- Alkaline Activator Preparation: Prepare an alkaline activator solution by mixing alkaline materials such as sodium hydroxide (NaOH) with a source of silica and alumina. Common sources of silica and alumina include sodium silicate (Na_2_SiO_3_). The proportions of the activator components depend on the desired geopolymer mix design.
- Mixing: Combine the processed agro waste powder with the alkaline activator solution. Mix them thoroughly until a homogeneous paste is formed. The mixing process can be done manually or by using mechanical mixing equipment.
- Molding: Pour or cast the geopolymer mixture into molds or formwork, similar to traditional concrete. Ensure proper compaction to eliminate air voids and achieve good consolidation of the mixture.
- Curing: Place the molded geopolymer concrete in a curing environment. Curing conditions may vary, but commonly the concrete is kept in a temperature-controlled environment (such as an oven or curing chamber) at a temperature around 60–80 °C. The curing period typically lasts for 24–48 h to promote geopolymerization and strength development.
- Demolding and Further Curing: After the initial curing period, remove the molds and allow the geopolymer concrete to further cure under ambient conditions or by providing additional moisture. This post-curing stage helps enhance the strength and durability of the concrete.

It is important to note that the specific proportions of agro waste, activator solution, and curing conditions may vary depending on the desired properties of the geopolymer concrete. Additionally, it is recommended to conduct laboratory tests and trials to optimize the mix design and fine-tune the process parameters for the specific agro waste materials being used^83,84^.

### The approach to designing mixes for geopolymer concrete

A straightforward mix design procedure is proposed for geopolymer concrete (GPC) using sugarcane bagasse ash (SCBA) and banana peel ash (BPA) as precursor, following the steps of the ACI 211^43^ specification design for cement concrete^85^.

**Step 1: Calculate target strength (F**_**t**_ **)**

Target strength in concrete mix design refers to the desired level of compressive strength that concrete should achieve after a specified curing period. This strength is essential because it ensures that the concrete meets the structural and durability requirements for its intended application. Achieving the target strength is crucial for the safety, performance, and longevity of the structure. Considering the aim of the research study the target compressive strength for SCBA-BPA precursor mix is obtained after 28 days of the oven (heat) and outdoor curing as shown in Eq. (6)^75^.

6 $$ \left( {{\text{F}}_{{\text{t}}} } \right) = {\text{f}}_{{{\text{ck}}}} + {1}.{\text{65 S}}_{{\text{d}}} $$

where f_t_ is the target average compressive strength of GPC at 28 days, f_ck_ is the characteristic compressive strength at 28 days and S_d_ is the Standard Deviation.

**Step 2: Choice of the slump:**

The choice of slump depends on the type of work and the appropriate value can be assumed according to workability requirement.

**Step 3: Approximate air content**

The approximate air content for GPC is an important factor influencing its properties, especially workability and durability. Approximate air content in SCBA-BPA blended GPC is also taken at 1% by volume of low calcium precursor GPC.

**Step 4: Selection of binder proportion and alkaline/binder ratio**

The binder proportion selection is primarily influenced by the required compressive strength and workability of the concrete. Workability is a critical parameter that varies with binder proportions. The alkaline solution/binder ratio, defined as the mass of the alkaline solution (NaOH + Na_2_SiO_3_) to the mass of the precursor binder (SCBA + BPA) in a GPC mix, significantly impacts the concrete's quality and compressive strength. A lower alkaline/binder ratio results in a stiffer mix with reduced compressive strength. Experimental studies have shown that increasing the alkaline/binder ratio enhances compressive strength. For instance, at a ratio of 0.45, the mix was stiff, whereas at 0.60, it tended to segregate. Given that different binder contents, aggregate-binder ratios, aggregate sizes, and other characteristics can produce varying compressive strengths under outdoor and oven curing conditions, it is preferable to establish the relationship between strength and alkaline-binder ratio for specific in situ conditions^84^.

**Step 5: Selection of aggregate/binder ratio for the required target strength (for SCBA + BPA)**

Based on experimental findings from relevant literatures, it's noted that higher aggregate-binder ratios lead to reduced compressive strength, resembling trends seen in regular concrete. The choice of aggregate-binder ratio is influenced by strength needs, binder quantity, and alkaline-binder ratio. In geopolymer concrete (GPC) formulations incorporating agro wastes (SCBA and BPA) with varying binder levels, the disparities in compressive strength are minimal, indicating that aggregate content has negligible impact on high-strength GPC^85^.

**Step 6: Selection of binder content for the required target strength**

The choice of binder content should ensure that the desired strength is achieved with the maximum binder content. It is also influenced by the proportion of sugarcane bagasse ash (SCBA) and banana peel ash (BPA), as well as the alkaline-binder ratio. The amount of binder needed to achieve the same compressive strength may vary depending on the degree of SCBA or BPA and their physicochemical properties. Moreover, curing temperature affects binder content requirements. Therefore, selecting the appropriate binder content depends on factors such as strength requirements, availability of source materials, workability, and curing conditions^86^.

**Step 7: Estimation of coarse aggregate (CA) and fine aggregate (FA) content**

**Figure 4:**
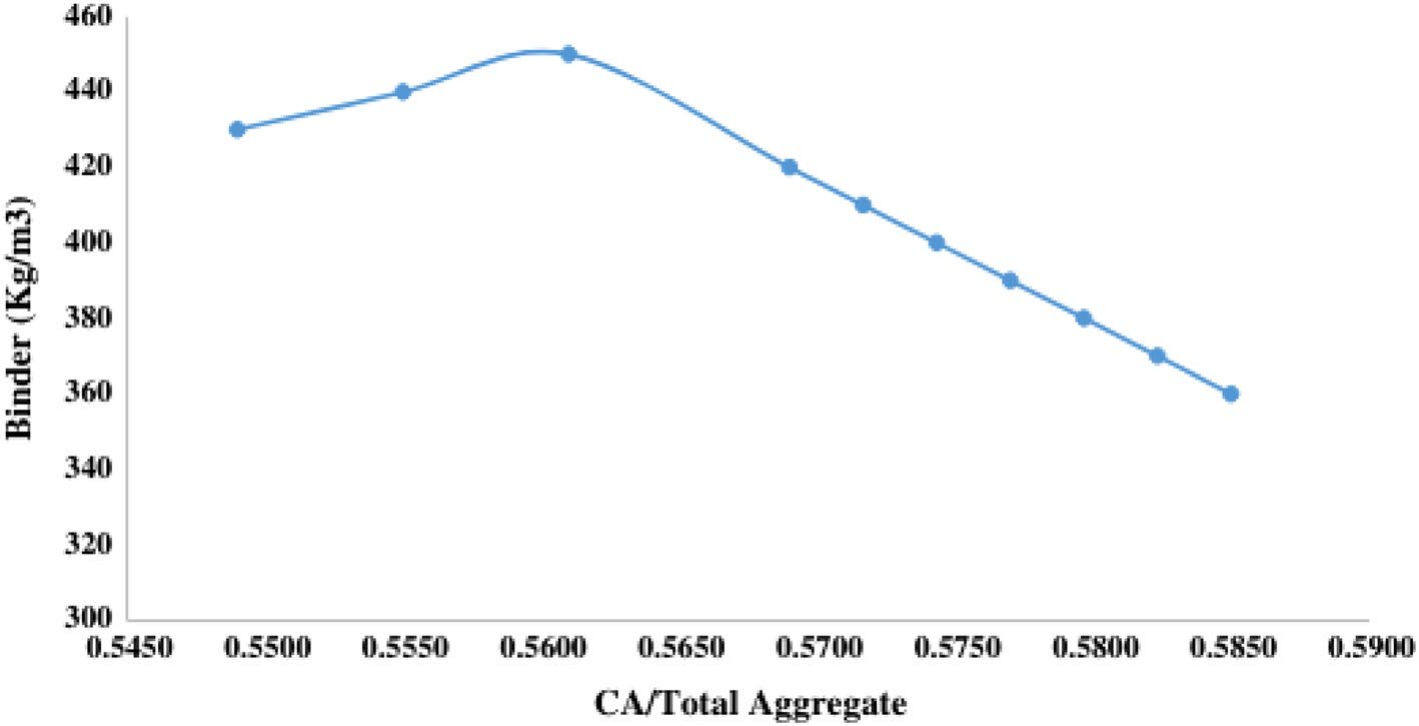
Binder content Vs coarse aggregate-total aggregate ratio for (360–450 kg/m^3^)^85^.

Figure 4 illustrates the variation of binder content with the coarse aggregate (CA) to total aggregate ratio. Through this graphical presentation, the CA to total aggregate ratio in the geopolymer concrete can be estimated for different selected binder contents. Based on the obtained ratios, the amounts of coarse aggregate and fine aggregate can be calculated using the expression in Eqns. 7–8^86,87^.

7 $$ {\text{Quantity of CA}} = \left( {{\text{CA}}/{\text{Total aggregate ratio}}} \right) \times {\text{Total aggregate content}} $$

8 $$ {\text{Quantity of FA}} = {\text{Total aggregate}} - {\text{CA}} $$

**Step 8: Determine alkaline content**

After determining the quantities of the alkaline activator solution and binder content, the amounts of coarse and fine aggregates can be calculated. Based on literature and experimental findings, the optimal mass ratio of Na₂SiO₃ solution to NaOH solution is established as 2.5. Consequently, the required quantities of Na₂SiO₃ and NaOH solutions are determined using the formula shown in Eqs. (9–11). Additionally, considering the strength and workability requirements, the molarity of NaOH is set at 10 M.

9 $$ {\text{Alkaline solution }} = {\text{ NaOH }} + {\text{ Na}}_{{2}} {\text{SiO}}_{{3}} $$

10 $$ {\text{Na}}_{{2}} {\text{SiO}}_{{3}} /{\text{NaOH solution}} = {2}.{5} $$

11 $$ {\text{Na}}_{{2}} {\text{SiO}}_{{3}} = {2}.{\text{5 NaOH}} $$

#### Illustration of mix proportions utilizing the suggested method

The mix design of geopolymer concrete (GPC) is detailed below, serving as an exemplar for the proposed methodology. Assume that geopolymer concrete made with SCBA and BPA is designed for a target strength of 30 MPa at 28 days with a slump of 50 mm. The resulting consistency and compressive strength suggest that the concrete has lower strength and reduced workability. Based on key details from relevant literature on design specifications, for a target compressive strength of 30 MPa, the total binder content is set at 375 kg/m^3^^75,84,86^. The alkaline-binder ratio is chosen as 0.55, leading to total aggregate-binder ratio of 5.02. The optimal dosage of Na₂SiO₃/NaOH is set at 2.5, using a 10 M NaOH solution.

To determine the quantity of the alkaline activator solution, the total binder content is multiplied by the ratio of the alkaline activator solution to the binder content = 375 kg/m^3^ × 0.55 = 206.25 kg/m^3^.

With the Na₂SiO₃/NaOH ratio of 2.5 known, the individual quantities of each alkaline solution can be calculated as shown:

$$ \begin{gathered} {\text{Solution of NaOH}} = {2}0{6}.{25/3}.{5} = {58}.{\text{93 kg/m}}^{{3}} \hfill \\ {\text{Solution of Na}}_{{2}} {\text{SiO}}_{{3}} = {58}.{93} \times {2}.{5} = {147}.{\text{33 kg/m}}^{{3}} \hfill \\ \end{gathered} $$

To determine the aggregate content for the geopolymer concrete mix, we multiply the obtained total aggregate to binder ratio of 5.02 by the total binder content (375 kg/m^3^):

$$ {\text{Total aggregate content }} = { 5}.0{2 } \times {\text{ 375 kg/m}}^{{3}} = {1882}.{\text{5 kg/m}}^{{3}} . $$

Total aggregate content = 5.02 × 375 kg∕m^3^ = 1882.5 kg∕m^3^.

To calculate the coarse aggregate (CA) content, we apply Eq. 7 by multiplying the Coarse aggregate/Total aggregate ratio (0.582) which Is derived from Fig. 4 by the calculated total aggregate content:

$$ {\text{CA}} = {1882}.{5} \times 0.{582} = {1}0{95}.{\text{62 kg/m}}^{{3}} . $$

To calculate the fine aggregate (FA) content, we apply Eq. 8 by subtracting the coarse aggregate content calculated from the total aggregate content:

$$ {\text{FA }} = { 1882}.{\text{5 kg/m}}^{{3}} - {1}0{95}.{\text{62 kg/m}}^{{3}} = {786}.{\text{88 kg/m}}^{{3}} $$

Table 5 presents the mix proportions for geopolymer concrete (GPC) designed to achieve a compressive strength of 30 MPa, cured at elevated temperatures (oven curing) and outdoor curing. Using these proportions, concrete cubes measuring 150 × 150 × 150 mm were cast and subjected to the specified curing method. The cubes were tested at various ages and under the two different curing conditions, with the obtained experimental results detailed in Table 6. The compressive strength results indicate that strength development significantly slowed after 28 days of curing. The variation between the target strength achieved for these mixes and the analytically developed results is less than 5% for all mixes. Therefore, the developed table is effective for all the proposed mixes under both outdoor and oven curing conditions for the specified target compressive strength. Specifically, for in situ conditions, the proposed mixes under outdoor conditions yield precise results^85,86^.

**Table 5:** Mix proportion of 30 MPa grade SCBA-BPA-GPC.

GPC ingredients	Quantity (kg/m^3^)
Precursor (SCBA + BPA)	375
Alkaline activator solution	206.25
NaOH	58.93
Na_2_SiO_3_	147.33
Coarse aggregate (CA)	1095.62
Fine Aggregate (FA)	786.88

**Table 6:** Average compressive strength results curing for 30 MPa concrete.

Curing age (days)	Average compressive strength (oven curing) (MPa)	Average compressive strength (outdoor curing) (MPa)
1	25.89	18.78
3	28.14	20.62
7	29.95	24.03
28	32.76	30.11

## Conclusion

In conclusion, the proposed simplified approach for designing geopolymer concrete mixtures offers a structured and accessible framework for engineers and practitioners involved in concrete production. By emphasizing key principles and optimizing material selection, mixture proportions, and testing protocols, this approach seeks to streamline the design process while maintaining the performance and sustainability benefits of geopolymer concrete.

- The approach includes steps such as selecting suitable raw materials, determining the alkaline activator solution, and adjusting mix proportions based on desired concrete properties. It also incorporates considerations for curing methods, testing procedures, and quality control measures.
- The determining factors (including chemical composition, alkali activation solution, water content, and curing conditions) of geopolymer are highly dependent on the source material utilized.
- The application of heat curing restricts the practical usage of geopolymer concrete, mainly confining its use to precast concrete applications.
- The expense associated with synthesizing geopolymer concrete using sodium silicate is comparatively high.
- This study proposes a novel simplified mix design for geopolymer concrete (GPC), drawing from the principles of Portland cement concrete (PCC) mix design outlined in ACI 211 (2009), along with recommended molar ratios of oxides involved in geopolymer synthesis. This streamlined approach aims to optimize the key factors influencing geopolymer concrete to achieve optimal compressive strength while maintaining acceptable workability. This method leverages commonalities between PCC and GPC, particularly in terms of water and aggregate.
- Future research should explore the potential substitution of sodium silicate with amorphous silica sources such as silica fume, rice husk ash, or ground waste glass in the activator solution to mitigate production costs.

Overall, the proposed methodology aims to facilitate the adoption of geopolymer concrete in construction projects by providing a practical and user-friendly guide. By promoting the use of alternative binders and sustainable materials, this approach contributes to the advancement of environmentally friendly building practices and helps address challenges associated with traditional cement-based concrete production. Further research and development in this area are crucial for optimizing mix designs, refining manufacturing processes, and promoting widespread adoption of geopolymer concrete technologies.

### Recommendations for future study

For future work on the proposed simplified approach for designing geopolymer concrete mixtures, several recommendations can be made to further enhance its effectiveness and applicability:

- **Experimental Validation**: Conduct experimental studies to validate the proposed methodology under various environmental conditions, aggregate types, and curing regimes. This will provide empirical data to support the accuracy and reliability of the approach.
- **Optimization of Mix Proportions**: Explore optimization techniques to refine the mix proportions and achieve the desired performance characteristics of geopolymer concrete. This may involve investigating the effects of different activator concentrations, curing temperatures, and aggregate types on the properties of the concrete.
- **Life Cycle Assessment**: Perform a comprehensive life cycle assessment (LCA) to evaluate the environmental impact of geopolymer concrete produced using the proposed approach compared to traditional cement-based concrete. This will help quantify the potential environmental benefits and identify areas for improvement.
- **Field Applications**: Apply the proposed methodology in real-world construction projects to assess its practical feasibility and performance in diverse applications. Field trials can provide valuable insights into the challenges and opportunities associated with implementing geopolymer concrete on a larger scale.
- **Standardization and Guidelines**: Work towards the development of standardized guidelines and specifications for geopolymer concrete mix design based on the proposed approach. Collaboration with industry stakeholders and regulatory bodies will be essential to ensure widespread adoption and acceptance of geopolymer concrete technologies.
- **Education and Training**: Provide education and training programs to engineers, contractors, and other stakeholders to familiarize them with the principles and practices of geopolymer concrete production. This will help build capacity and foster innovation in the construction industry.

By addressing these recommendations, future research on the proposed simplified approach for designing geopolymer concrete mixtures can contribute to the advancement and adoption of sustainable construction practices while minimizing the environmental impact of concrete production.

### Consent to participate

All authors were highly cooperative and involved in research activities and preparation of this article.

## Data Availability

All data generated or analyzed during this study are included in this published article.
