# Author Correction: Proposed simplified methodological approach for designing geopolymer concrete mixtures

**DOI:** 10.1038/s41598-024-69791-9

**Published:** 2024-08-29

**Authors:** George Uwadiegwu Alaneme, Kolawole Adisa Olonade, Ebenezer Esenogho, Mustapha Muhammad Lawan

**Affiliations:** 1 Civil Engineering Department, Kampala International University, Kampala, Uganda; 2 Department of Electrical, Telecommunication and Computer Engineering, Kampala International University, Kampala, Uganda; 3 Department of Electrical Engineering Science (Centre for Telecommunication), University of Johannesburg, Auckland Park, South Africa; 4 Department of Electrical Engineering, University of Botswana, Gaborone, Botswana

Correction to: *Scientific Reports* 10.1038/s41598-024-66093-y, published online 02 July 2024

The original version of this Article omitted an affiliation for Ebenezer Esenogho. The correct affiliations are listed below:

Department of Electrical, Telecommunication and Computer Engineering, Kampala International University, Uganda.

Department of Electrical Engineering Science (Centre for Telecommunication), University of Johannesburg, Auckland Park, South Africa.

Department of Electrical Engineering, University of Botswana, Gaborone, Botswana.

The original Article has been corrected.

